# Cryoballoon-Assisted Pulmonary Vein Isolation and Left Atrial Roof Ablation Using a Simplified Sedation Strategy without Esophageal Temperature Monitoring: No Notable Thermal Esophageal Lesions and Low Arrhythmia Recurrence Rates after 2 Years

**DOI:** 10.3390/diagnostics14131370

**Published:** 2024-06-27

**Authors:** Damir Erkapic, Konstantinos Roussopoulos, Marko Aleksic, Korkut Sözener, Karel Kostev, Josef Rosenbauer, Samuel Sossalla, Dursun Gündüz, Joachim Labenz, Christian Tanislav, Kay Felix Weipert

**Affiliations:** 1 Department of Cardiology, Rhythmology and Angiology, Medical Clinic II, Diakonie Klinikum Jung Stilling, 57074 Siegen, Germany; konstantinos.roussopoulos@diakonie-sw.de (K.R.); marko.aleksic@diakonie-sw.de (M.A.); korkut.soezener@prontomail.com (K.S.); josef.rosenbauer@diakonie-sw.de (J.R.); dursun.guenduez@diakonie-sw.de (D.G.); kay.weipert@diakonie-sw.de (K.F.W.); 2 Department of Cardiology and Angiology, Medical Clinic I, University Hospital Giessen, 35392 Giessen, Germany; samuel.sossalla@innere.med.uni-giessen.de; 3 Department of Epidemiology, Philipps-University Marburg, 35037 Marburg, Germany; karel.kostev@iqvia.com; 4 Department of Gastroenterology, Medical Clinic I, Diakonie Klinikum Jung Stilling, 57074 Siegen, Germany; joachim.labenz@diakonie-sw.de; 5 Department of Geriatrics and Neurology, Diakonie Klinikum Jung Stilling, 57074 Siegen, Germany; christian.tanislav@diakonie-sw.de

**Keywords:** atrial fibrillation, cryoballoon, ablation, pulmonary vein isolation, roof line, LARA, EDEL, esophageal lesion

## Abstract

Background: Atrial fibrillation (AF) ablation is increasingly effective for managing heart rhythm but poses risks like esophageal fistulas. Minimizing esophageal thermal lesions while simplifying procedures is crucial. Methods: This prospective study involved 100 consecutive AF patients undergoing cryoballoon ablation with simplified sedation, without esophageal temperature monitoring. Patients with paroxysmal AF (Group A) received pulmonary vein isolation only, while those with persistent AF (Group B) also had left atrial roof ablation. Gastroesophageal endoscopy was performed post-procedure to detect lesions, and cardiological follow-ups were conducted at 3, 12, and 24 months. Results: The cohort included 69% men, with a median age of 65.5 years. Post-ablation endoscopy was performed in 92 patients; esophageal lesions were found in 1.1% of Group A and none of Group B. GERD was diagnosed in 14% of patients, evenly distributed between groups and not linked to lesion occurrence. Gastric hypomotility was observed in 16% of patients, with no significant difference between groups. At 24 months, arrhythmia-free survival was 88% in Group A and 74% in Group B. Conclusion: Cryoballoon-assisted pulmonary vein isolation, with or without additional left atrial roof ablation and without esophageal temperature monitoring during a simplified sedation strategy, shows low risk of esophageal thermal injury and effective ablation outcomes.

## 1. Introduction

As atrial fibrillation (AF) ablation gains widespread acceptance for its efficacy in rhythm control, cryoballoon ablation is progressively emerging as a primary therapeutic approach [1,2,3]. Beyond pulmonary vein isolation, some authors highlight additional efficacy advantages through the performance of left atrial roof ablation, referred to as LARA [4,5,6,7]. The increasing use of the cryoballoon technique—even beyond pulmonary vein isolation (PVI)—requires a comprehensive investigation of its safety profile, especially with regard to esophageal lesions. The anatomic proximity of the left atrium to the esophagus during atrial fibrillation ablation procedures poses a potential risk of thermal injury to the esophagus. Studies have shown that up to 22% of atrial fibrillation patients may develop esophageal thermal lesions (EDELs) following cryoballoon PVI [8,9]. This can lead to a serious complication known as an esophagoatrial fistula, which unfortunately has a high mortality rate of up to 90% following conservative management. Mortality also remains high even after surgical (52%) or endoscopic (57%) treatment [10]. The occurrence of esophagoatrial fistulas has recently been reported to be as frequent as 0.038% with radiofrequency (RF) ablations and 0.0015% with cryoballoon procedures, and indeed, it is likely that there are more cases that go unreported [10]. Our investigation focuses on assessing the incidence of thermal esophageal lesions after PVI and in particular beyond PVI, where the cryoballoon is used without an esophageal temperature probe. Furthermore, arrhythmia-free survival was assessed during mid-term follow-up under these circumstances.

## 2. Methods

### 2.1. Study Design and Study Population

The present study constitutes a subgroup analysis of a prospective observational study. The detailed protocol and methodology of the study have been published previously [11].

**Figure 1:**
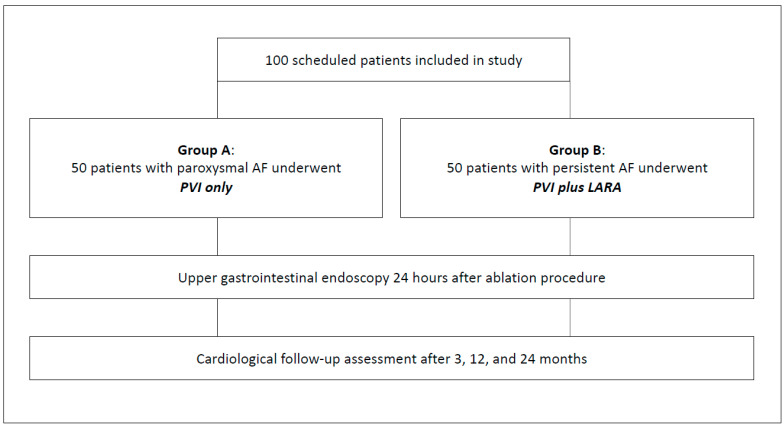
Study flowchart. AF = atrial fibrillation; PVI = pulmonary vein isolation; LARA = left atrial roof ablation.

In brief, one hundred consecutive patients were enrolled in this study, which was approved by the Ethics Committee of the University of Münster, Germany (AZ 2019-779-f-S). All patients received left atrial ablation treatment for the first time. Group A comprised 50 patients with paroxysmal AF, while Group B included 50 patients with persistent AF. Group A underwent pulmonary vein isolation (PVI) only, whereas Group B received a combined treatment of PVI and an additional left atrial roof ablation (LARA) using the cryoballoon technique (Figure 1). All procedures were performed under conscious sedation with diazepam (Ratiopharm^®^, Ulm, Germany) and piritramide (Hameln^®^, Hameln, Germany) without the use of an esophageal temperature probe. The administration of these drugs was standardized, starting with 7.5 mg of piritramide and 5 mg of diazepam intravenously prior to groin puncture, followed by repetitive bolus doses of 3.75 mg of piritramide and 2.5 mg of diazepam as needed. A sufficient level of sedation was defined and pursued as one in which the patient did not perceive the procedure as uncomfortable. This was ensured through communication between the nursing staff and the patient as well as monitoring of patients’ vital signs. Where external electrical cardioversion was required at the end of procedure, supplementary administration of etomidate (B. Braun, Melsungen AG, Melsungen, Germany) at a dosage of 0.05–0.1 mg/kg/body weight was carried out.

The main purpose of the present cohort study was to investigate the burden of endoscopically detected esophageal lesions (EDELs) after PVI only (Group A) and PVI plus LARA (Group B) without the use of an esophageal temperature probe.

Endoscopic examinations, conducted by highly experienced gastroenterologists, were carried out on all patients 24 h post-ablation treatment. Esophageal lesions were classified based on the Novel Kansas City classification [12]. As part of the gastroscopy, additional pathologies potentially associated with AF or with the ablation procedure, such as gastric hypomotility and/or gastroesophageal reflux disease (GERD), were naturally identified. Gastric hypomotility was defined as retention of food in the stomach after an overnight fast >16 h [13,14]. Stages of GERD were assessed using the Los Angeles Classification [15].

### 2.2. Atrial Fibrillation Ablation Procedure

All examinations were performed using a biplane fluoroscopy system (Siemens Artis Zee biplane, Erlangen, Germany) in RAO 30° and LAO 60° views. After transseptal puncture using an SL-1 sheath and a BRK-1 needle (Abbott, St. Paul, MN, USA), angiography of the left atrial and pulmonary veins was performed. Immediately prior to angiography, rapid pacing with a cycle length of 300 ms was applied through a decapolar catheter (ViaCath™, Biotronik, Berlin, Germany) in the right ventricle. Subsequently, 15 mL of contrast agent was injected manually into the left superior pulmonary vein (LSPV) followed by the right superior pulmonary vein (RSPV) via the SL-1 sheath. The contrasted borders of the left atrium, pulmonary veins, and transseptal puncture site were then traced on the examiner’s monitor using a board marker to ensure safe movement and positioning of the cryoballoon during the ablation procedure. All ablations were performed exclusively using the 4th-generation cryoballoon Arctic Front Advance Pro 28 mm (Medtronic Inc., Mounds View, MN, USA) following the replacement of the SL-1 sheath with the CryoAdvance sheath (Medtronic Inc., Mounds View, MN, USA) using the Seldinger technique. Pulmonary vein signals were mapped with an inner lumen spiral mapping catheter (Achieve catheter™, 20 mm diameter, Medtronic Inc.) before, during, and after each cryoenergy application. Guided by the Achieve catheter™, the 28 mm cryoballoon was advanced through the sheath into the left atrium, inflated proximally to the pulmonary vein ostium, and then gently pushed to occlude the pulmonary vein. Vessel occlusion and atrial regurgitation were assessed by selectively injecting contrast medium via an automatic injection pump (CVi^TM^, ACIST Europe B.V., Heerlen, The Netherlands) at a standardized volume of 5 mL, flow of 3 mL/s, and pressure of 350 psi. After achieving the best possible occlusion, a 240 s freeze–thaw cycle was performed. We chose to implement a 240 s freezing cycle for all pulmonary veins because, based on our experience and that of some other research groups, a freeze duration of 240 s compared to 180 s increased lesion durability [16]. We have not observed an increased complication rate as a result. However, we perform all our atrial fibrillation ablation procedures under conscious sedation without the use of an esophageal probe. If a temperature of −60 °C was reached before the completion of the 240 s freeze–thaw cycle, the freeze–thaw time was terminated earlier. The freeze–thaw cycle was also interrupted if sufficiently low temperatures (≤40 °C) could not be achieved after 60 to max. 180 s. In these cases, the balloon was repositioned, followed by a second freeze. During the ablation of the septal pulmonary veins, the decapolar catheter was positioned in the superior vena cava for ipsilateral phrenic nerve stimulation. Phrenic nerve function was monitored using diaphragmatic compound motor action potentials (CMAPs). Ablation was stopped immediately if substantial decreases in CMAPs were observed, indicating weakening or loss of diaphragmatic contraction. Successful isolation of the pulmonary veins was defined by demonstrating the elimination of pulmonary vein potentials measured at the ostium of each pulmonary vein using a 20 mm Achieve catheter™ (Medtronic) as well as demonstrating an entry and exit block via the Achieve catheter™ and the decapolar CS-catheter (ViaCath™).

**Figure 2:**
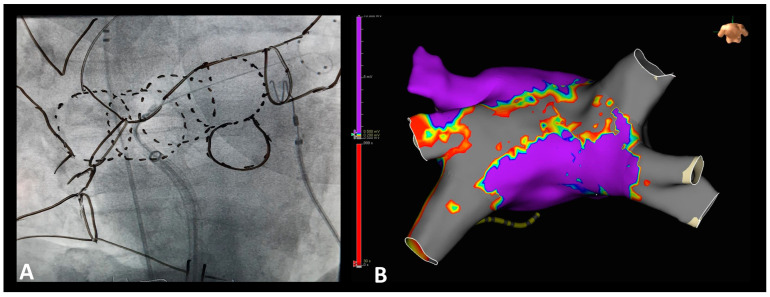
(**A**) Left atrial roof ablation (LARA) technique using cryoballoon ablation in RAO 30° view. The borders of the left atrium, pulmonary veins, transseptal puncture site, and overlapping freezes were traced on the examiner monitor using a board marker; (**B**) 3D representation of LARA in the Voltage MAP (threshold 0.5/0.2 mV) during AF Redo procedure in follow-up using the EnSite Precision Cardiac Mapping System (Abbott). RAO = right anterior oblique; AF = atrial fibrillation.

Additional left atrial roof ablation (LARA) was conducted in all patients with persistent AF. Sequential, overlapping 180 s freezes were applied along the left atrial roof, starting near the position used for left superior pulmonary vein isolation. This was performed using a gentle clockwise rotation combined with gentle sheath retraction and incremental advancement of the cryoballoon until the position used for right superior pulmonary vein isolation was reached (Figure 2A,B). The Achieve catheter™ was anchored in the left superior pulmonary vein for all cryoballoon positions at the left atrial roof. In case of ongoing intraprocedural AF, electrical cardioversion (200 Joule biphasic AP) was performed, followed by verification of pulmonary vein isolation and a conduction block at the left atrial roof, as previously describe d [6]. In brief, the Achieve catheter™ was positioned at the caudal and cranial posterior LA wall, guided by the steerable sheath. Subsequently, baseline pacing during sinus rhythm at the right atrial upper septum with a cycle length of 500 ms was performed. Activation times at the caudal and cranial positions of the posterior LA wall next to the LA roof were measured. A caudocranial ascending activation at the posterior LA wall and a conduction delay of more than 120 ms next to the LARA verified the conduction block of the LA roof [4,5]. Touch-up ablation with RF was not performed, even if the PV was not isolated or the LA roof line was not complete.

Echocardiography was performed immediately after ablation to rule out pericardial effusion. After removal of the catheters and venous sheaths, the access site was closed with subcutaneous temporary purse-string sutures [17]. Patients were then monitored telemetrically for 24 h.

After ablation, all patients received the proton pump inhibitor pantoprazole as standard. This was administered prophylactically at double the standard dose for 2 weeks, followed by a single dose for a further 4 weeks.

Cardiology follow-up examinations were performed at 3, 12, and 24 months after the procedure, each including a detailed medical history, physical examination, echocardiography, resting ECG, and 24 h Holter electrocardiograms.

In addition, a final telephone contact with each patient was made at the end of the follow-up period. Antiarrhythmic drugs (classes I and III) were stopped 3 months after ablation. Arrhythmia-free survival was defined as the absence of documented episodes of atrial fibrillation, atrial flutter, or atrial tachycardia lasting more than 30 s after an initial 90-day blanking period, and patients reporting no further sensations of cardiac arrhythmias.

### 2.3. Statistical Analysis

Categorical variables were reported as frequencies and percentages. The normal distribution was assessed using Kolmogorov–Smirnov’s one-sample test. Nonparametric data were analyzed using a two-tailed Mann–Whitney U-test. The Yates Chi-Square test was utilized to compare relative frequencies. For presenting proportions of patients free of arrhythmia in the follow-up time, survival curves were calculated and plotted as a graph (patients with persistent versus paroxysmal AF were compared). Statistical analysis was conducted using SPPS software (version 22.0. IBM Corporation, Armonk, NY, USA).

## 3. Results

Patient characteristics and details of the ablation procedure are presented in Table 1.

The 240 s freeze–thaw cycle was stopped earlier in 66 of 390 PVs due to (1) reaching the target safety temperature at 45 PVs during PVI (10 LSPV, 5 LIPV, 18 RSPV, 12 RIPV), (2) lack of sufficiently low temperatures during PVI (14 PVs: 6 LSPV, 1 LIPV, 3 RSPV, 4 RIPV), requiring the balloon to be repositioned for a second freeze, and (3) substantial decreases in CMAPs at septal PVI in 13 PVs (9 RSPV, 4 RIPV). Among these, four patients experienced transient phrenic nerve injuries, which fully healed within 24 h to 3 months. Cryoballoon-energy delivery during LARA did not need to be stopped early at any time in any of the 50 patients with persistent atrial fibrillation. The freezing times in each PV as well as in LARA are listed in Table 1.

Gastroesophageal endoscopy was performed 24 h after ablation in 92 of 100 patients. Four patients from Group A and four patients from Group B declined to undergo post-interventional gastroscopy.

**Figure 3:**
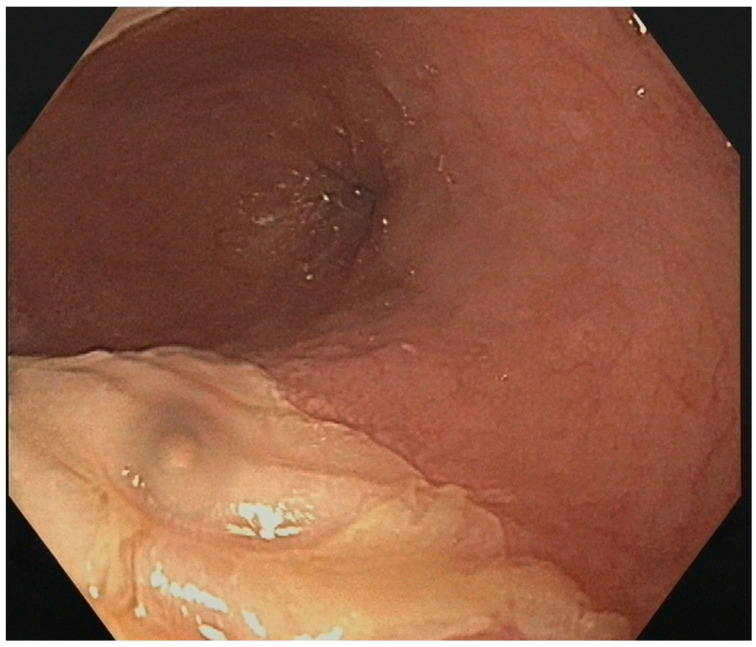
Endoscopically detected esophageal superficial lesion after PVI only.

Ablation-related esophageal lesions were identified in only 1 of the 92 patients. This manifested as a superficial ulcer measuring approximately 2 cm and corresponding to a type 2a lesion under the Novel Kansas City Classification. The lesion was located in the mid-esophagus region, approximately 28 cm from the dental arch, retrocardiac at the anterior wall of the esophagus (Figure 3). This event was observed in Group A (Table 2) and remained clinically silent. Additional gastroesophageal reflux disease (GERD) as a comorbidity could not be identified in this patient. Compared to the overall study cohort, the patient with the observed superficial ulcer had slightly lower temperatures in most pulmonary veins: LSPV −52 °C vs. −48 °C, RSPV −56 °C vs. −53 °C, and LIPV −47 °C vs. −46 °C. Conversely, temperatures of −43 °C were measured at the patient’s right inferior pulmonary vein (RIPV), compared to −51 °C in the overall study cohort. The LA index was 30.8 mL/m^2^ compared to the median 39 mL/m^2^ (IQR 29.9–50.9) in the total study group. Furthermore, the patient’s BMI was 27.1, compared to a median BMI of 28.45 kg/m^2^ in the overall patient population.

No ablation-related esophageal lesions were observed in Group B. Incidentally, gastric hypomotility was evident in 15 out of 92 patients (16%). Gastric hypomotility was clinically silent in 14 of these 15 patients (93%). One patient from Group B (7%) developed symptomatic gastric hypomotility, necessitating treatment with a prokinetic agent for three months (Prucalopride 1 mg once daily, Aristo Pharma GmbH, Germany). Over this period, symptoms gradually diminished, and the patient had achieved complete resolution of symptoms after three months. There was no statistically significant gastric hypomotility in either of the two groups (Group A 15.2% vs. Group B 17.4%, *p* = 0.777). In addition, other pathologies such as gastroesophageal reflux disease were more frequently observed endoscopically in both groups than was initially known from the patients’ history: GERD was present in 12/92 patients (14%), 6 patients (14%) in each group, *p* > 0.999. The initial diagnosis of GERD was made in 8/87 patients (9%) during post-procedural gastroscopy (*n* = 4 in each group, *p* = 0.973) (Table 2).

**Figure 4:**
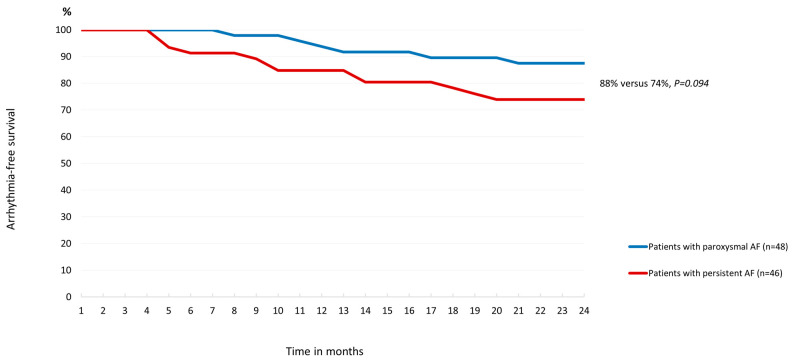
Arrhythmia-free survival at mid-term follow-up after PVI only and PVI+LARA using the cryoballoon.

A mid-term follow-up of 24 months was completed by 48/50 patients in Group A and 46/50 patients in Group B. Six patients (6%) were lost in mid-term follow-up. Arrhythmia-free survival was 88% in Group A and 74% in Group B (Figure 4). Atrial fibrillation was the cause of recurrences in 66.6% of cases (4 of 6 patients in Group A and 8 of 12 in Group B), while 33.4% were attributed to atypical atrial flutter or atrial tachycardia. Atrial tachycardia was observed in 16.6% of cases: two patients in Group A and one patient in Group B. Perimitral atrial flutter occurred in three patients (16.6%), all in Group B. Overall, there were no cases of roof-dependent atrial flutter as a cause of recurrence.

All patients with recurrence except for three patients with AF recurrence underwent redo ablation. These three patients initially favored medication-based antiarrhythmic therapy over a 3D-redo procedure.

Roof line blockage failed with the cryoballoon during the first procedure in three patients. Therefore, preserved LA roof conduction could be naturally observed in these patients during 3D-redo. In another four patients with an initially proven LA roof conduction block after LARA, roof conduction recovery could be demonstrated during the 3D-redo procedure. Finally, persistent roof line blockage created by the cryoballoon was observed in only two of the six patients with initial successful LARA.

## 4. Discussion

The main finding of this prospective observational study demonstrates that despite the absence of esophageal temperature monitoring in cryoballoon ablation procedures extending beyond the pulmonary veins, no notable thermal esophageal lesions were detected while using a simplified conscious sedation strategy.

Sarairah et al. reported EDELs after PVI using the cryoballoon only in 22% of 95 patients studied [8]. Obesity, atrioesophageal distance, and thinner anterior esophageal wall thickness—as measured by pre-procedural magnetic resonance imaging—were identified as independent risk factors for EDELs. All ablations in their study were performed under general endotracheal anesthesia using an ETP. Fürnkranz et al. observed EDELs in 19% of 32 study participants [9]. They recognized a significant correlation between a decrease in esophageal temperature measured with ETP and the occurrence of EDELs. They suggested that esophageal temperatures falling below 12 °C during cryoballoon ablation were predictive of esophageal injury with a sensitivity of 100% and specificity of 92%. All procedures were performed under sedation with boluses of midazolam, fentanyl, and continuous infusion of propofol. The authors concluded that low esophageal temperature measurement accurately predicts lesion formation and may improve the safety of PVI with the second-generation cryoballoon.

Osorio et al. performed a study of 30 patients with cryoballoon PVI and additional left atrial posterior wall (LAPW) ablation, monitored with an ETP [18]. After the ablation procedures, gastroscopy performed 24 h later revealed the absence of EDELs in all patients. The study was performed under general anesthesia, and cryoenergy delivery was terminated prematurely in 17% of cases due to the measurement of temperatures below 15 °C by the ETP. By contrast, Shigeta et al. reported an incidence of an EDEL in 10.9% of patients during cryoballoon PVI and LAPW in a significantly larger case series of 101 patients, despite the use of an ETP [19]. Cryofreezing applications were interrupted prematurely in 48.5% of patients during the left atrial bottom line of LAPW due to esophageal temperatures < 15 °C. All procedures were performed under continuous infusion of propofol in combination with dexmedetomidine hydrochloride.

Our study, which included endoscopic examinations of 92 patients, showed a low incidence of esophageal injury (1.1%), even with the addition of extrapulmonary cryoballoon ablation in the absence of an ETP. In the patient population studied, only one patient in the PVI-only group had a superficial and clinically silent lesion in the esophagus. Based on the observations of Sarairah et al. [8], our patient with an EDEL also had obesity (BMI 27.1 kg/m^2^). However, compared to the entire population in our study with a median BMI of 28.45 kg/m^2^, no other patient showed an injury to the esophagus. Whether there was an additional shorter distance between the esophagus and the left atrium and/or a thinner anterior wall of the esophagus cannot be determined from our study because no MRI examination was performed.

Compared to other published studies, pulmonary vein isolation in our study was performed on patients in a sufficiently but not too deeply sedated state with bolus administrations of dipidolor and diazepam. This approach may contribute to less restriction of esophageal motility as a protective reflex against thermal effects compared to deeper sedation, potentially influencing the occurrence of EDELs [20]. While deeper sedation is typically required with the use of an ETP in terms of patient tolerance, it is not necessarily required without the use of such a probe. PVI is known to be associated with a higher risk of esophageal thermal injury in patients under general anesthesia than in those under conscious sedation [21]. While temperature monitoring has been shown to be beneficial for patients under deep sedation or even general anesthesia, it can be omitted when patients are not under excessively deep sedation. This “keep it simple” technique might simplify the ablation procedure for both the patient and the electrophysiologist without increasing the safety risk to the esophagus, as shown in our study. On the other hand, the supposedly protective use of an ETP during catheter-based atrial fibrillation ablation must also be critically evaluated. In both atrial fibrillation ablation studies using radiofrequency and using cryoballoon techniques, the use of ETPs is described to be associated with an increased risk of EDELs [14,22]. Miyazaki et al. used an ETP in 40 patients undergoing second-generation CB ablation and none in 64 patients [14]. Pulmonary vein isolation was performed with one 28 mm balloon using a single 3 min freeze technique. The use of ETP was observed as a predictor of EDELs with a hazard ratio of 15.750; 95% confidence interval 1.887–131.471; and *p* = 0.011. The study group speculated that the use of an ETP during cryoablation of the LIPV could cause the esophagus to become wedged between the balloon anteriorly and the thoracic spinal column or aorta posteriorly, increasing the likelihood of injury. In our study, no significant EDEL rate was observed in the absence of an ETP and with simultaneous superficial depth of sedation. Ultimately, it cannot be determined from our study design whether the depth of sedation alone, the non-use of an esophageal temperature probe, or the combination of both is responsible for the low esophageal lesion rate observed in our study. Finally, a randomized multi-arm study is needed to clarify this point.

Due to the close proximity of the esophagus and the left atrium, some studies have suggested an association between gastroesophageal reflux disease (GERD) and atrial fibrillation [19,23,24]. In our study, we did not observe a significant difference in the prevalence of GERD between the two groups, with at least 86% showing no endoscopic evidence of GERD.

In our study, gastric hypomotility was found in 16% of all cases, with no significant difference between the PVI-only group and the PVI plus LARA group. In a study by Shigeta et al., which included 54 patients with persistent AF, a lower LA roof (LA 5.7 (5.1–6.1) mm vs. 8.8 (7.1–11.2) mm, *p* < 0.001) was observed as a predictor of gastric hypomotility when performing LARA [25]. The cutoff LA roof height for predicting gastric hypomotility after LARA was 6.1 mm, with a sensitivity of 85% and specificity of 83%. They reported gastric hypomotility in 18.5% of all cases. However, LARA was performed in all patients, so there was no control group with PVI only. Miyazaki et al. reported gastric hypomotility after PVI only in 17.3% of cases [14]. Therefore, it is not certain whether additional CB ablation of the left atrial roof increases the risk of gastric hypomotility. Our data at least showed no significant difference between the two groups, although it must be noted that the height of the left atrium as a potential predictor was not measured.

The benefit of ablation beyond the pulmonary veins in patients with AF is controversial. While a prospective randomized multicenter trial using RF energy showed no extra benefit of additional posterior wall isolation, two meta-analyses of 10 and 26 studies, respectively, and including up to 3287 patients with AF, did find evidence of a clear benefit [26,27,28]. In patients with persistent AF, the adjunctive posterior wall isolation (PWI) was associated with significantly reduced recurrence rates of all atrial arrhythmias (risk ratio: 0.74; *p* < 0.001) and AF specifically (risk ratio: 0.67; *p* = 0.01), particularly in older patients and/or those with a larger left atrial diameter [26]. Adjunctive PWI, whether using radiofrequency or the cryoballoon, reduced AF recurrence, with radiofrequency showing a trend toward higher recurrence rates of atrial tachycardia and/or atrial flutter [26]. In our study, PWI was not performed, only LARA. However, it has been reported that LA substrate modification by LARA is beneficial for improving outcomes in patients with persistent atrial fibrillation [4,7]. Nevertheless, our study was not designed primarily to demonstrate the efficacy of adding LARA. The freedom from recurrence rates observed during the median follow-up period of our study—74% for persistent atrial fibrillation—are comparable to those described in the literature [4,7]. Kuniss et al. reported arrhythmia-free survival rates in 57% of patients with PVI only and 75.6% of those with PVI plus LARA in 399 patients with persistent AF [4].

## 5. Limitations

Since we did not perform a direct comparison in this study between procedures with and without an esophageal temperature probe, or between general anesthesia and conscious sedation, definitive proof of the safety benefits of the ablation procedure performed with a simplified sedation strategy without the use of an esophageal temperature probe remains to be determined. A larger randomized study on this topic is needed to support the results of this study.

## 6. Conclusions

Cryoballoon-assisted pulmonary vein isolation, with or without additional left atrial roof ablation, and without the use of an esophageal temperature probe, in combination with a simplified conscious sedation strategy, was not associated with an increased risk of esophageal thermal injury or reduced ablation efficacy in the present non-randomized study.

## Figures and Tables

**Table 1:** Baseline and procedural ablation characteristics.

Baseline Characteristics	Total (*n* = 100)	Paroxysmal AF (*n* = 50)	Persistent AF (*n* = 50)	*p*
**Age** (median, IQR *) (years)	65.5 (58.2–72.4)	66.1 (57.3–71.4)	65.5 (58.8–74.4)	0.564
**Sex**				
Male	69 (69%)	28 (56%)	41 (82%)	0.009
Female	31 (31%)	22 (44%)	9 (18%)	
**BMI** (median, IQR *), (kg/m^2^)	28.5 (25.5–33.4)	27.4 (23.9–31.8)	30.3 (26.7–34.7)	0.016
**CHA2DS2VASc** (median, IQR *, range)	2 (1–3, 0–7)	2 (1–3)	2 (1–4)	0.056
**Echocardiography**				
Left atrium index (mL/m^2^) (median, IQR *)	39.0 (29.9–50.9)	31.7 (24.3–38.9)	48.1 (38.5–56.1)	<0.001
Mitral insufficiency I–II° (median, IQR *, range) (*n* = 77)	I° (I°–I°; I°–II°)	I° (0–I°)	I° (I°–I°)	0.037
Left ejection fraction (median, IQR *, range) (%)	60 (55–60; 40–75)	60 (60–65)	55 (50–60)	<0.001
**Comorbidities**				
Hypertension	71 (71%)	33 (66%)	38 (76%)	0.387
Diabetes mellitus	18 (18%)	7 (14%)	11 (22%)	0.378
Coronary artery disease	21 (21%)	10 (20%)	11 (22%)	0.999
Sleep apnea	11 (11%)	3 (6%)	8 (16%)	0.201
Heart insufficiency				
NT-Pro-BNP (ng/L) (median, IQR) (*n* = 61)	417.0 (86.5–153.5)	232.5 (138.3–470.5)	742.0 (206.5–2178.5)	0.004
Kidney disease				
eGFR (mL/min/1.73 m^2^) (median, IQR)	76.0 (64.4–88.9)	77.5 (66.8–89.1)	75.2 (58.4–89.0)	0.295
Gastroesophageal reflux disease	5 (5%)	3 (6%)	2 (4%)	0.999
Previous stroke	8 (8%)	2 (4%)	6 (12%)	0.269
**Medication Prior to Procedure**				
Intake of oral anticoagulants	95 (95%)	45 (90%)	50 (100%)	0.056
Intake of platelet inhibitors	7 (7%)	1 (2%)	6 (12%)	0.112
Intake of proton pump inhibitors	25 (25%)	12 (24%)	13 (26%)	>0.999
**Procedural Ablation Characteristics**				
**Pulmonary Vein Isolation**				
Acute ablation success	100 (100%)	50 (100%)	50 (100%)	0.999
Total number of freezes (median, IQR *)	5 (4–5)	5 (4–5)	5 (4–6)	0.172
Cryoenergy application total time (min) (median, IQR *)	16.2 (15.1–20.0)	16.0 (15.2–19.5)	17.1 (15.0–22.1)	0.435
RSPV Cryoenergy application time (min) (median, IQR *)	4 (3–4)	4 (3–4)	4 (3–5)	0.811
RIPV Cryoenergy application time (min) (median, IQR *)	4 (4–4)	4 (4–5)	4 (4–4)	0.698
LSPV Cryoenergy application time (min) (median, IQR *)	4 (4–4)	4 (4–4)	4 (4–4)	0.944
LIPV Cryoenergy application time (min) (median, IQR *)	4 (4–4)	4 (4–4)	4 (4–4)	0.347
Nadir temperature (°C) (median, IQR *)				
Nadir temperature RSPV (°C) (median, IQR *)	−53 (−56 to −49)	−54 (−56 to −51)	−52 (−56 to −48)	0.326
Nadir temperature RIPV (°C) (median, IQR *)	−51 (−55 to −47)	−52 (−55 to −47)	−50 (−54 to −47)	0.323
Nadir temperature LSPV (°C) (median, IQR *)	−48 (−53 to −45)	−48 (−54 to −44)	−49 (−53 to −46)	0.218
Nadir temperature LIPV (°C) (median, IQR *)	−46 (−51 to −45)	−46 (−48 to −45)	−48 (−54 to −45)	0.062
**Left Atrial Roof Ablation (LARA)**				
Acute ablation success			41 (82%)	
Total number of freezes (median, IQR *, range)			4 (3–4; 3–6)	
Cryoenergy application time (min) (median, IQR *)			9 (9–18)	
Nadir temperature (°C) (median, IQR *)			−40 (−33 to −46)	

Adapted and modified from Erkapic et al., ‘*Diagnostics*’ (2023) [11]. AF = atrial fibrillation; IQR = interquartile range; eGFR = estimated glomerular filtration rate; BMI = body mass index; PV = pulmonary vein; RSPV = right superior pulmonary vein; RIPV = right inferior pulmonary vein; LSPV = left superior pulmonary vein; LIPV = left inferior pulmonary vein; * = Q1/Q3.

**Table 2:** Gastroesophageal pathologies diagnosed 24 h after PVI only and beyond.

	Total (*n* = 92)	Paroxysmal AF (*n* = 46)	Persistent AF (*n* = 46)	*p*
**GERD**	13 (14%)	6 (13%)	7 (15%)	0.765
**Los Angeles Classification**				
Grade A: mucosal break ≤ 5 mm	12 (13%)	6 (13%)	6 (13%)	
Grade B: mucosal break >5 mm	1 (1%)	0 (0%)	1 (2%)	
Grade C: mucosal break involving <75% of esophageal circumference	0 (0%)	0 (0%)	0 (0%)	
Grade D: mucosal break involving ≥75% of esophageal circumference	0 (0%)	0 (0%)	0 (0%)	
**EDEL**	1 (1%)	1 (2%)	0 (0%)	>0.999
**Novel Kansas City Classification**				
Type 1: erythema	0 (0%)	0 (0%)	0 (0%)	
Type 2a: superficial ulcer	1 (1%)	1 (2%)	0 (0%)	
Type 2b: deep ulcer	0 (0%)	0 (0%)	0 (0%)	
Type 3a: perforation without communication with the atria	0 (0%)	0 (0%)	0 (0%)	
Type 3b: perforation with atrioesophageal fistula	0 (0%)	0 (0%)	0 (0%)	
**Gastric Hypomotility**	15 (16%)	7 (15%)	8 (17%)	0.777

PVI = pulmonary vein isolation; GERD = gastroesophageal reflux disease; EDEL = endoscopically detected esophageal lesion.

## Data Availability

The data presented in this study are available on request from the corresponding author. The data are not publicly available due to privacy and ethical restrictions.
